# Launching *eLife*, Part 2

**DOI:** 10.7554/eLife.00365

**Published:** 2012-12-13

**Authors:** Randy Schekman, Fiona Watt, Detlef Weigel

**Keywords:** publishing, peer review, open access, eLife

## Abstract

With a commitment to open access and innovation in peer review, *eLife* aims to publish important results in the life and biomedical sciences in a flexible digital format that allows authors to present their work in full, including the key data on which the conclusions are based.

**Figure 1:**
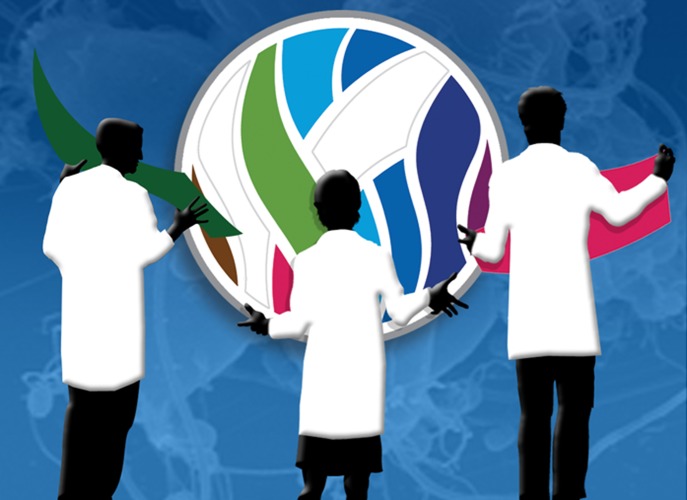
IMAGE: WWW.N-DESIGN.CO.UK

The first papers to be accepted by *eLife* have been available on PubMed Central (PMC) since 15 October. At the time we wrote: “But it is at the *eLife* journal website where we will have the most scope to experiment and explore new territory in research communication” (Schekman et al., 2012). The *eLife* journal website is now live and, at the time of writing, includes a total of 19 research articles (including 12 that have already been released at PMC), plus a variety of other content. Going forward, all new content will be published on the *eLife* journal website as well as at PMC (and its mirror sites, such as Europe PMC).

*eLife* has its origins in a conversation between the leaders of the Wellcome Trust, the Max Planck Society and the Howard Hughes Medical Institute (HHMI), who all felt it was time for funding agencies and scientists to work together to take a leading role in the publication of research results, and to break down the pay walls that restricted access to these results. As a result of these discussions, *eLife* has been founded on three basic principles: the results of scientific research should be freely available to everyone, to read and to use; decisions about the fate of submitted papers should be fair, constructive, and provided in a timely manner; and the presentation of new research findings needs to take full advantage of all the possibilities offered by digital media and platforms.

Although open access has received a great deal of attention, and a degree of acceptance by some subscription publishers, only about 10% of the biomedical literature is published in this format. Moreover, authors have few options if they want to publish a paper under an open-access licence in a highly selective journal. This is one of the reasons why the Wellcome Trust, the Max Planck Society and HHMI decided to fund a highly-selective, open-access journal to publish outstanding papers in the life and biomedical sciences, irrespective of their funding source.

*eLife* opened for submissions several months ago and the response has been most encouraging, with authors, editors and referees all embracing our fresh approach to the review process. This process starts with Senior Editors, often in consultation with specialists on our Board of Reviewing Editors (BRE), making the initial decision—within a few days—as to whether a manuscript will be sent out for full review. If a manuscript is considered a potentially important contribution, a member of the BRE is asked to serve as the reviewing editor and to select one or two additional referees. After the referee reports are returned, the reviewing editor and the referees confer in an online consultation session to decide if the manuscript represents a significant advance in the field and if it meets our high standards of technical excellence. If the decision is favourable, the authors receive a decision letter listing the points that need to be addressed in the revised version of the paper: authors are not sent all the referee reports in their entirety and do not, therefore, have to respond to redundant or conflicting comments in the reports. If the reviewing editor and referees find the work is too limited or technically too weak to be revised without major additional work, the paper is rejected.

We find this consultation session greatly facilitates the decision process and allows the reviewing editor to provide the authors with a decision letter that contains a clear, coherent statement of what is required to publish the work in *eLife*. The reviewing editor, having served as a referee and conferred with the other referee(s) on the decision letter, is then in a position to make a decision to accept—or not—a revised paper, generally without having to consult the referee(s) again. This streamlined process, which depends crucially on the participation of active scientists in all aspects of the review and editorial process, reduces the time it takes to make a final decision.

Once accepted, *eLife* papers are quickly processed and published in both HTML and PDF formats. Each article has an *eLife* digest that explains the work, and its background, to a general readership, and selected articles are accompanied by an Insight article written by an expert in the field (sometimes the reviewing editor or one of the referees). In the PDF version of the research articles, we have adopted a somewhat unusual one-column format, which we feel makes the article easier to read on screens and, in particular, on portable devices. Each element in the article (including the abstract, digest, figures, tables and so on) has its own digital object identifier (DOI), which allows the elements to be cited (or linked to) directly, and will help readers to search for specific information within an article. And if the authors agree, the HTML version includes the initial decision letter and the authors' response to it.

Because *eLife* is an online-only journal, there are no artificial limits on the number of pages, figures or references in research articles. As such we are able to link additional data to the primary text, to integrate videos and other rich media, and to associate extra figures with each primary figure, rather than locking away valuable additional information in a large file of supplementary or supporting information. Our intent is to improve the integrity of published work and to facilitate the use and re-use of the narrative and data by readers. To this end, we are also making article XML available via sites such as github and Fluidinfo, where researchers and programmers will be able to access and interact with *eLife* content in new and experimental ways. We will monitor the success of these and all other aspects of *eLife* (such as our media policy, which does not involve the use of embargoes), over the coming months as we continue to experiment and learn.

At *eLife*, our goal is to place science and scholars first. We are not in the business of selling magazines, so neither profits nor fashions drive our decisions. Furthermore, with the support of our funders, we are able to waive any publication charges for our first three years of operation. We hope you enjoy the new *eLife* website and these first articles, and we invite you to choose *eLife* as a preferred venue for the publication of your best work.
